# Development and Explainable Machine Learning Validation of a Novel Sleep Disturbance Ratio for Obstructive Sleep Apnea Severity Assessment

**DOI:** 10.3390/diagnostics16162606

**Published:** 2026-08-17

**Authors:** Mehmet Kabak, Halit Irmak, Abdullah Reşit Kılıç, Barış Çil

**Affiliations:** 1 Department of Chest Disease, Mardin Artuklu University, Mardin 47200, Turkey; 2 Department of Artificial Intelligence, Faculty of Computer Engineering, Mardin Artuklu University, Mardin 47200, Turkey; halitirmak@artuklu.edu.tr; 3 Department of Chest Disease, Mardin Training and Research Hospital, Mardin 47100, Turkey; abdullahresit@icloud.com (A.R.K.); drbariscil@hotmail.com (B.Ç.)

**Keywords:** obstructive sleep apnea, machine learning, explainable artificial intelligence, sleep disturbance ratio, polysomnography, sleep architecture, XGBoost, Random Forest

## Abstract

**Background/Objectives**: Obstructive sleep apnea (OSA) is traditionally classified according to the apnea–hypopnea index (AHI), although AHI alone does not fully capture the heterogeneity of disease severity. This study introduces a novel polysomnography-derived biomarker, the Sleep Disturbance Ratio (SDR), and evaluates its contribution to OSA severity classification using explainable machine learning approaches. **Methods**: A retrospective study was conducted using polysomnographic data from 767 adults who underwent overnight sleep studies. SDR was calculated as the logarithmic ratio between light sleep (N1 + N2) and restorative sleep (N3 + REM). Predictive models were trained and evaluated using four established machine learning algorithms: Decision Tree, Random Forest, Extreme Gradient Boosting (XGBoost), and Artificial Neural Network (ANN). Model performance was assessed using accuracy, Cohen’s kappa, F1-score, multiclass AUC, ROC analysis, feature importance ranking, partial dependence plots, and clinical risk mapping. **Results**: Although SDR did not differ significantly across conventional OSA severity groups in univariate analysis (*p* = 0.77), explainable machine learning analyses consistently demonstrated that increasing SDR was associated with a higher probability of severe OSA, particularly in combination with lower mean oxygen saturation. SDR also showed strong physiological relevance by correlating positively with N2 sleep (r = 0.85) and negatively with N3 sleep (r = −0.83), supporting its role as a biomarker of sleep fragmentation. Among the predictive models, Random Forest achieved the highest classification accuracy (75.0%), whereas XGBoost demonstrated the best multiclass discrimination (AUC = 0.895) and the highest ROC performance for severe OSA (AUC = 0.962). ESS remained the most influential predictor across all models. **Conclusions**: This study introduces SDR as a novel polysomnography-derived biomarker that captures sleep architecture disruption beyond conventional AHI-based assessment. Although SDR is not an independent diagnostic marker, explainable machine learning analyses demonstrated that it provides complementary physiological information for OSA severity classification, particularly when integrated with oxygenation parameters.

## 1. Introduction

Obstructive Sleep Apnoea Syndrome (OSAS) is a common and significant public health problem characterised by recurrent upper airway obstructions during sleep. The condition is associated with cardiovascular diseases, metabolic disorders, neurocognitive dysfunction and increased mortality via intermittent hypoxia, sleep fragmentation, changes in intrathoracic pressure and activation of the sympathetic nervous system [1]. Current epidemiological data suggest that approximately one billion adults worldwide may be affected by OSAS to varying degrees, and it is reported that the global burden of the condition is steadily increasing in line with rising obesity prevalence [1].

Whilst polysomnography (PSG) is the gold standard for the diagnosis of OSAS, the Apnoea–Hypopnoea Index (AHI) is traditionally used to determine the severity of the condition. Although the AHI reflects the number of apnoeas and hypopnoeas per hour, studies conducted in recent years have shown that this parameter cannot fully account for the biological and clinical heterogeneity of the condition [2,3]. Significant differences may be observed in terms of symptom burden, cardiovascular risk, neurocognitive impairment and quality of life among individuals with similar AHI values [3,4]. Although the Apnoea–Hypopnoea Index (AHI) remains the gold standard for diagnosing and classifying OSA, it primarily quantifies the frequency of respiratory events and does not fully capture their physiological consequences on sleep architecture. Patients with similar AHI values may exhibit substantial differences in sleep fragmentation, oxygen desaturation, daytime sleepiness, and overall clinical burden. These observations have highlighted the need for complementary polysomnographic biomarkers capable of reflecting dimensions of OSA beyond respiratory event frequency alone.

It is therefore thought that assessing OSAS solely on the basis of respiratory event counts may be insufficient, and that complementary biomarkers are required. In recent years, it has been suggested that measures such as hypoxic burden, oxygen desaturation profiles, the arousal index and sleep architecture parameters may be more valuable than AHI in predicting the clinical outcomes of OSAS [5,6]. In particular, it has been shown that a reduction in deep sleep (N3) and REM sleep, coupled with an increase in light sleep stages, is associated with cardiometabolic risk, daytime symptoms and quality of life [7]. These changes in sleep architecture reflect not only the respiratory but also the neurophysiological burden of the condition.

Sleep fragmentation is recognised as one of the key components of the pathophysiology of OSAS. As a result of recurrent respiratory events and micro-awakenings, the proportion of light sleep stages increases, whilst there is a marked reduction in the restorative N3 and REM sleep stages [8]. However, there are currently only a limited number of standardised and easily interpretable composite biomarkers that comprehensively represent this imbalance. Therefore, the development of new parameters capable of quantitatively characterising macrostructural changes in sleep architecture is of importance from both a clinical and a research perspective.

Advances in artificial intelligence and machine learning algorithms have opened up new opportunities for the analysis of high-dimensional biomedical data [9]. In particular, algorithms such as Random Forest, XGBoost and artificial neural networks have demonstrated successful results in the diagnosis and severity classification of OSAS, thanks to their ability to model complex non-linear relationships between variables [10]. However, the vast majority of existing studies have focused directly on AHI-based classification, whilst the use of interpretable and physiologically meaningful biomarkers that reflect sleep architecture has remained quite limited.

With the increasing use of machine learning in the healthcare sector, ensuring that the decision-making mechanisms of models are understandable has also become a key requirement. Consequently, explainable artificial intelligence (XAI) approaches have come to the fore in recent years [11]. XAI methods enhance clinicians’ trust in AI-based systems and support clinical applicability by demonstrating which variables contribute to model predictions and to what extent [12].

This study describes the Sleep Disturbance Ratio (SDR), a new biomarker developed using sleep stage percentages derived from polysomnography data. The SDR is a new parameter designed to quantitatively express the balance between superficial sleep stages (N1 + N2) and restorative sleep stages (N3 + REM). The primary objective of the study is to assess the relationship between the SDR and the severity of OSAS, to compare the performance of different machine learning algorithms, and to examine the role of the SDR in model decision-making processes using explainable artificial intelligence methods. It is believed that this approach could contribute to a better understanding of the multidimensional nature of OSAS and to the development of more interpretable clinical decision support systems in the future.

## 2. Materials and Methods

### 2.1. Study Design and Patient Population

**Figure 1:**
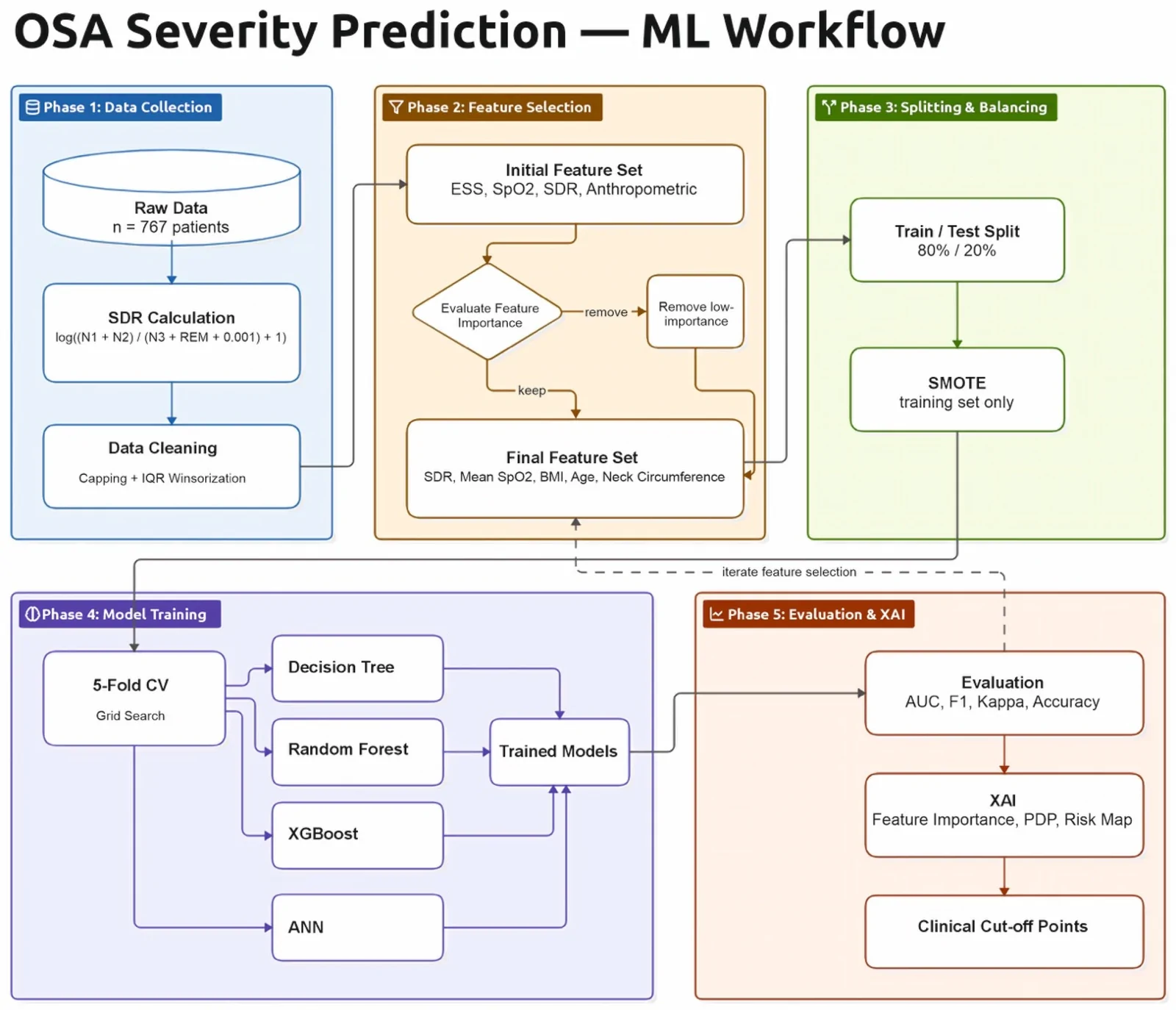
Overall workflow of the machine learning pipeline. After data collection and SDR calculation, preprocessing and feature selection were performed. The dataset was divided into training and testing subsets, SMOTE was applied only to the training data, and machine learning models were trained using five-fold cross-validation with hyperparameter optimization. Model performance was evaluated on the independent test set, followed by explainable artificial intelligence (XAI) analysis to interpret feature contributions. The blue boxes represent data processing stages, the yellow boxes denote the modelling phases, and the arrows indicate the sequential flow of the data pipeline.

The aim of this retrospective observational study was to predict the severity of Obstructive Sleep Apnoea Syndrome (OSAS) using machine learning (ML) algorithms. A total of 767 adult patients who had undergone a full-night polysomnography (PSG) examination in a sleep laboratory were included in the study (Figure 1). Polysomnography (PSG) was performed using a Philips Respironics Alice 6 polysomnography system (Philips Respironics, Mardin, Türkiye). Data preprocessing, machine learning modeling, and statistical analyses were performed using R software version 4.6.0. Consecutive patients included in the study were identified from institutional records, and the study was conducted as a retrospective analysis of existing data. The study was approved by the Mardin Artuklu University Non-Interventional Clinical Research Ethics Committee (meeting no. 7, decision no. 2026/7-27; 2 July 2026) and was conducted in accordance with the Declaration of Helsinki. Given the retrospective nature of the study and the use of existing data, the requirement for written informed consent was waived. All data were anonymized and analyzed in accordance with applicable ethical and data-protection requirements.

Demographic, anthropometric, clinical and polysomnographic data relating to patients were retrospectively obtained via the electronic patient record system. The dataset included anthropometric measurements such as age, sex, body mass index (BMI), neck circumference and waist circumference, as well as comorbid conditions such as hypertension, diabetes mellitus and chronic kidney disease.

Objective respiratory parameters derived from polysomnography recordings included mean oxygen saturation, minimum oxygen saturation and the percentage of time spent within the 80–90 per cent oxygen saturation range (S80–90). In addition, Epworth Sleepiness Scale (ESS) scores were used to assess subjective daytime sleepiness.

The Apnoea–Hypopnoea Index (AHI) was used as the outcome variable, and patients were classified into four groups according to the criteria of the American Academy of Sleep Medicine (AASM): Normal: AHI < 5; Mild OSAS: AHI 5–14.9; Moderate OSAS: AHI 15–29.9; Severe OSAS: AHI ≥ 30 [13].

### 2.2. Polysomnography Assessment

All patients underwent a standard full-night Type I polysomnography. The PSG recordings included electroencephalography (EEG), electrooculography (EOG), submental electromyography (EMG), electrocardiography (ECG), nasal airflow, thoracoabdominal respiratory movements and pulse oximetry parameters.

Sleep stages and respiratory events were assessed by experienced sleep specialists in accordance with the AASM scoring criteria. Apnoea was defined as a reduction in airflow of more than 90 per cent lasting ≥10 s, whilst hypopnoea was defined as a reduction in airflow of ≥30 per cent accompanied by oxygen desaturation [13].

### 2.3. Feature Engineering: Sleep Disruption Ratio

A new parameter has been developed with the aim of converting macro-structural changes in sleep architecture into a clinical biomarker. This value, obtained by dividing the total percentage duration of light sleep stages (N1 and N2) by that of deep sleep stages (N3 and REM), has been named the Sleep Disturbance Ratio (SDR). To correct for statistical skewness in the values and prevent asymptotic errors, a constant of 0.001 was added to the formulated equation, and by applying the natural logarithm, the parameter SDR = log(((N1 + N2)/(N3 + REM + 0.001)) + 1) was derived.

### 2.4. Data Pre-Processing and Class Imbalance Management

As part of the data pre-processing protocol, measurement errors were corrected by capping percentage values at 100. Outliers in numerical variables were suppressed using the Interquartile Range (IQR) method. However, the key clinical indicators determining disease severity (AHI and oxygen saturation data) were exempted from this process to enable the model to learn from outlier cases.

To verify the generalizability of the models on an independent test set, the dataset was partitioned at the subject level into an 80% training set and a 20% test set. Each participant contributed only a single polysomnography recording, and no subject appeared in both the training and test sets, thereby ensuring a subject-independent data split and preventing data leakage. To prevent sample imbalance between the dependent variable classes from adversely affecting model sensitivity, a synthetic data up-sampling method was applied only to the training set. The independent test set remained completely untouched throughout model development and was used exclusively for the final performance evaluation.

### 2.5. Algorithmic Modelling and Statistical Analysis

For the classification problem, the Decision Tree (DT), Random Forest (RF), XGBoost and Artificial Neural Network (ANN) algorithms were used. The models’ hyperparameters were optimised using 5-fold cross-validation and grid search methods. Performance evaluation utilised the metrics of accuracy, Kappa, F1-score and the multi-class Area Under the Curve (AUC). The effects of the independent variables on the model were examined using normalised (0–100) importance scores; the class-specific probability trends of the SDR parameter were analysed using Partial Dependence Plots (PDPs). The Kruskal–Wallis test was used for statistical group comparisons. In addition to the multi-class performance evaluation, ROC analyses were conducted using a one-versus-rest approach to examine the diagnostic discriminatory power for cases of severe OSAS (Class 3). ROC curves were generated for each machine learning model, and discriminatory performance was assessed using AUC values. A *p*-value of <0.05 was considered statistically significant in all statistical analyses.

## 3. Results

### 3.1. Exploratory Data Analysis and Correlation Structure

The correlation matrix, created to assess the relationships between clinical and polysomnographic parameters, showed that the strongest relationship was between the target variable, AHI, and the ESS score (r = 0.78). This finding supports the notion that subjective daytime sleepiness is strongly associated with the severity of OSAS.

**Figure 2:**
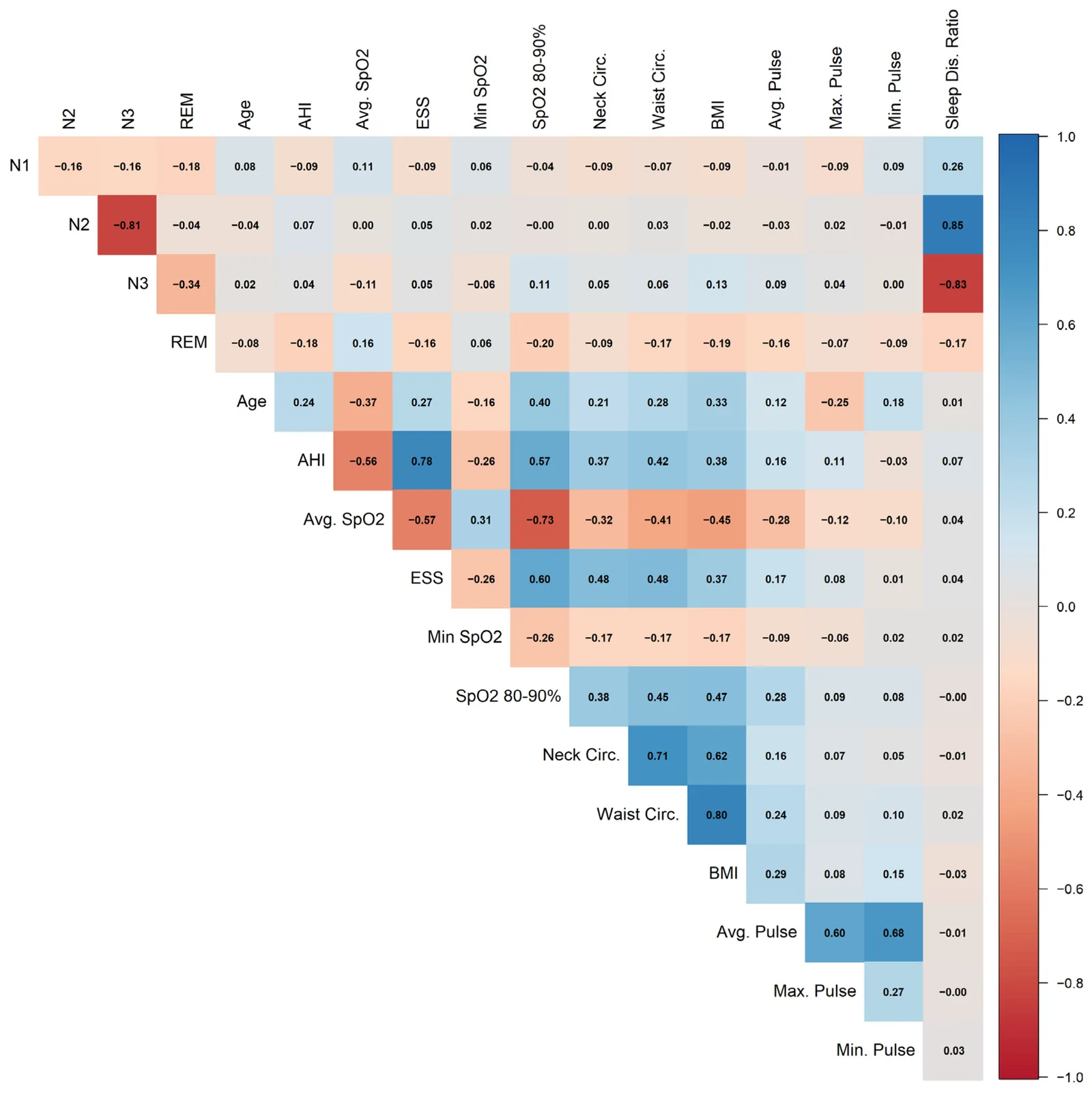
Hierarchical clustering correlation matrix of clinical variables.

Upon examination of the newly developed SDR parameter, it was observed that SDR showed a strong positive correlation with N2—one of the superficial sleep stages (r = 0.85)—whilst exhibiting a strong negative correlation with N3, the restorative deep sleep stage (r = −0.83). This correlation pattern suggests that SDR possesses physiological validity as a composite biomarker reflecting sleep fragmentation and loss of deep sleep (Figure 2).

### 3.2. Model Performance

The performance metrics of the machine learning models, trained using all clinical, anthropometric and questionnaire variables, on the test dataset are presented in Table 1.

Whilst the Random Forest model achieved the highest classification accuracy at 75 per cent, the XGBoost algorithm demonstrated the highest discriminative performance with a multi-class AUC value of 0.895. In contrast, the Artificial Neural Network model achieved lower accuracy (62.5 per cent) and F1-score (59.7 per cent) compared to the other algorithms.

The fact that the Kappa coefficients were at a medium-to-high level across all models (0.48–0.65) indicates that the models did not merely classify randomly and were able to distinguish between OSAS severity classes in a meaningful way.

These results demonstrate that ensemble-based algorithms (particularly Random Forest and XGBoost) offer more stable and robust performance in OSAS severity classification.

### 3.3. Attribute Importance Levels and ESS Dominance

Normalised variable importance scores were analysed in order to evaluate the models’ decision-making mechanisms.

In all algorithms, the ESS score ranked first with the maximum normalised importance value (100) and emerged as the dominant determinant in the model prediction processes. This finding suggests that subjective daytime sleepiness is one of the strongest indicators of OSAS severity, not only from a clinical perspective but also in terms of algorithmic classification.

Objective oxygenation parameters namely minimum saturation, mean saturation and the S80–90 percentage contributed significantly, particularly in the Decision Tree and Random Forest models. In contrast, the relative contribution of the SDR parameter remained at a lower level, varying between 6.5% and 23.6% across the algorithms.

Nevertheless, the fact that the contribution of SDR particularly in the Random Forest model is on a par with that of certain other anthropometric variables suggests that this new parameter holds potential as a complementary biomarker representing disruption in sleep architecture.

To evaluate the independent contribution of the remaining variables, a sensitivity analysis was performed after excluding the Epworth Sleepiness Scale (ESS) from the feature set (Table 2B). Across all machine learning models, the percentage of sleep time with SpO_2_ between 80% and 90% emerged as the most influential predictor. Oxygenation-related variables, including minimum and mean oxygen saturation, also remained among the leading predictors. Notably, the Sleep Disturbance Ratio (SDR) consistently ranked among the most important features across the Decision Tree, Random Forest, and XGBoost models, with normalized importance scores of 40.37, 54.99, and 38.15, respectively. These findings indicate that SDR retains meaningful predictive value even in the absence of ESS and supports its complementary role in OSA severity classification (Table 2B).

### 3.4. The Class-Based Statistical and Algorithmic Impact of SDR

The relationship between SDR and OSAS severity has been assessed using both classical statistical methods and model-based, explainable artificial intelligence analyses.

**Figure 3:**
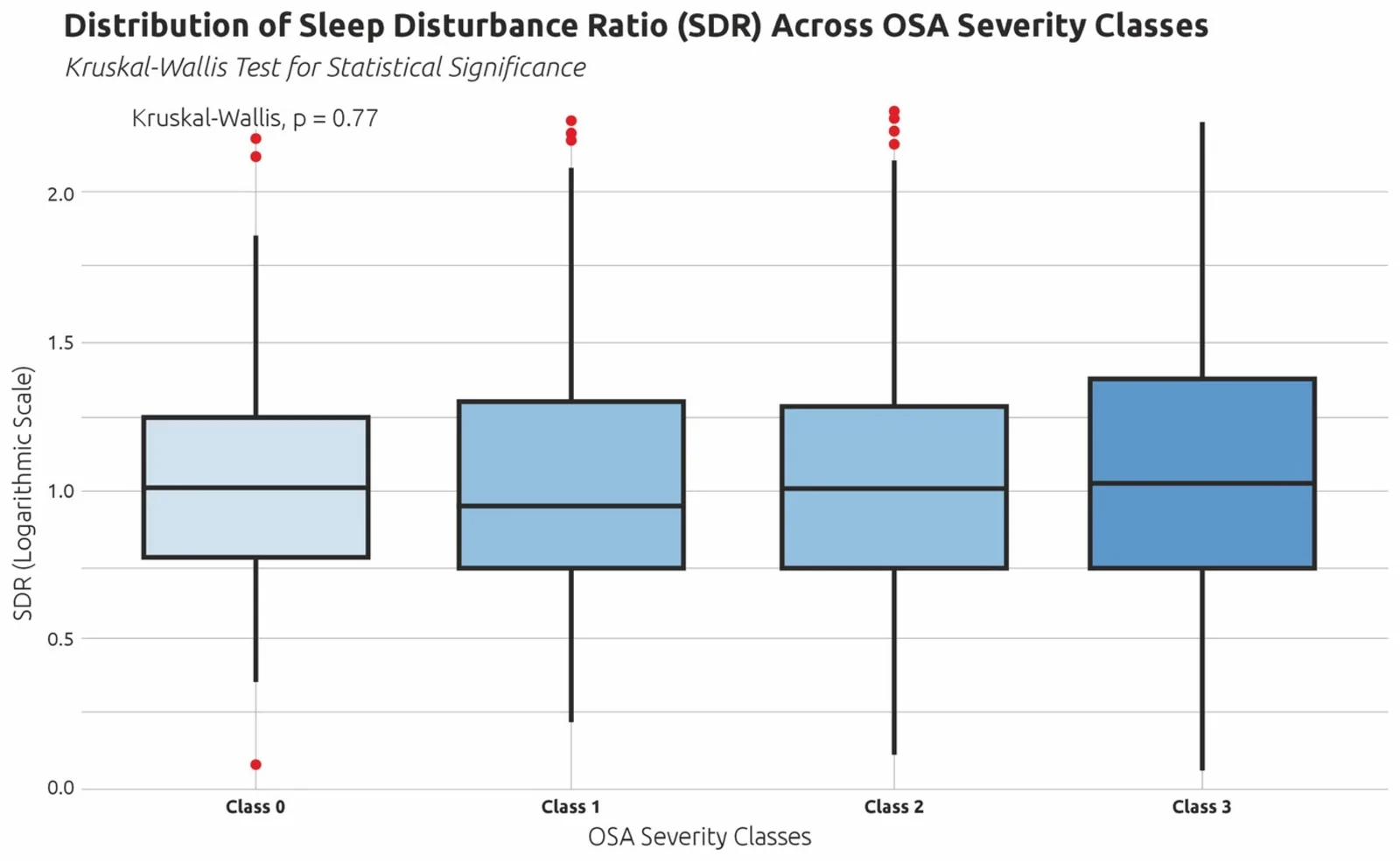
Distribution of SDR by OSAS Severity Classes and Statistical Analysis. Box plots represent the interquartile range, with the horizontal line indicating the median and whiskers indicating the range. Red dots indicate outliers. Different shades of blue represent the OSA severity classes. The Kruskal–Wallis test showed no statistically significant difference among the groups (*p* = 0.77).

The Kruskal–Wallis test revealed no statistically significant difference in median SDR values between the OSAS severity classes (*p* = 0.77). However, although there was no statistical significance in univariate analyses, it was observed that SDR could influence class probabilities in multivariate model-based analyses (Figure 3).

**Figure 4:**
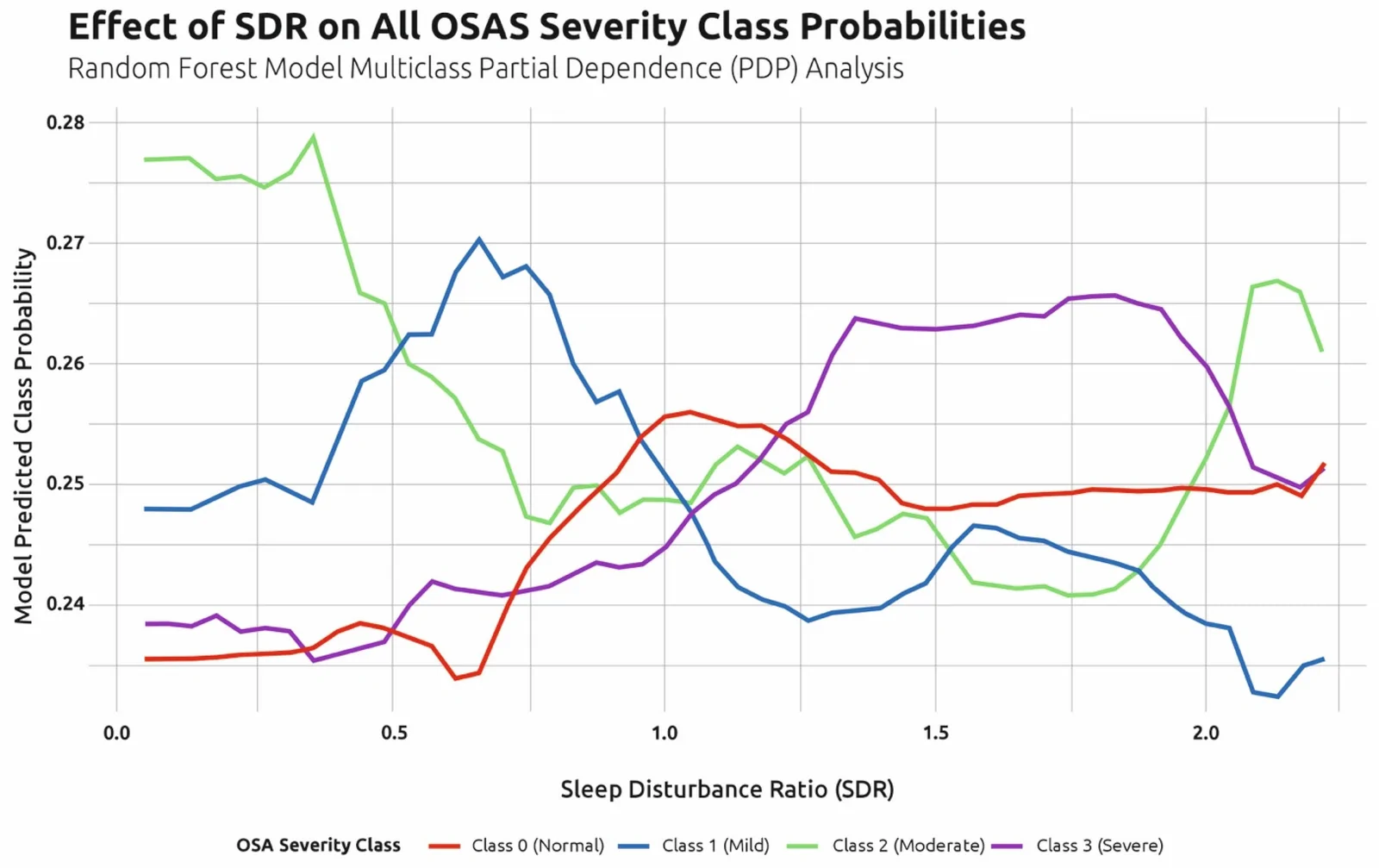
Partial Dependence Plot (PDP) Showing the Effect of the Sleep Disturbance Ratio (SDR) on the Predicted Probabilities of OSA Severity Classes.

As illustrated in Figure 4, the partial dependence plot (PDP) demonstrates that within the principal range of observed SDR values, increasing SDR is generally associated with a higher predicted probability of severe OSA (Class 3) and a lower predicted probability of mild OSA (Class 1). This relationship is non-linear and should not be interpreted as monotonic. The fluctuations observed at the extreme ends of the SDR distribution are likely attributable to the lower density of observations in these regions, a recognized characteristic of partial dependence analyses in tree-based ensemble models. Overall, the findings suggest that SDR contributes as a complementary biomarker to OSA severity classification when interpreted alongside other clinical variables, rather than as a standalone predictor.

### 3.5. Clinical Risk Map and Interaction Surface

The risk topography, created to assess the interaction between SDR and mean oxygen saturation, is presented in Figure 4.

In the heatmap analysis, it was observed that the likelihood of severe OSAS increases in regions where high SDR values co-occur with low mean saturation. However, due to the dominant influence of ESS in the model’s decision-making processes, it was found that the risk gradient resulting from the interaction between SDR and saturation is concentrated within a relatively narrow probability band.

This finding suggests that, rather than being a strong classifier on its own, SDR may function as a complementary parameter that reflects the multidimensional nature of OSAS severity when evaluated in conjunction with oxygenation parameters.

### 3.6. Comparison of ROC Curves for Machine Learning Models in the Diagnosis of Severe OSA

**Figure 5:**
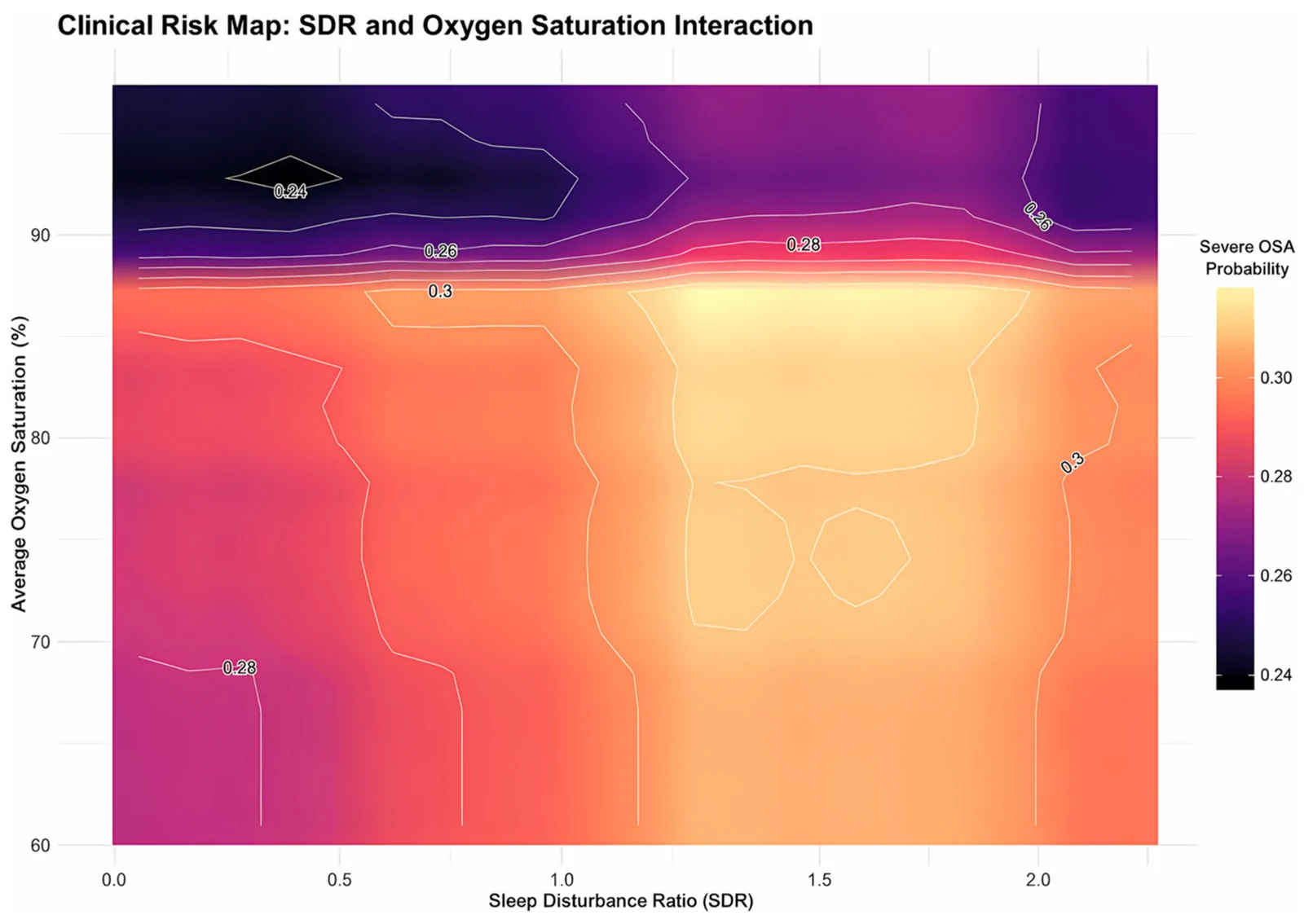
Clinical Risk Map: Interaction between SDR and Saturation. White contour lines indicate equal levels of severe OSA probability, with the corresponding probability values labeled on the lines.

**Figure 6:**
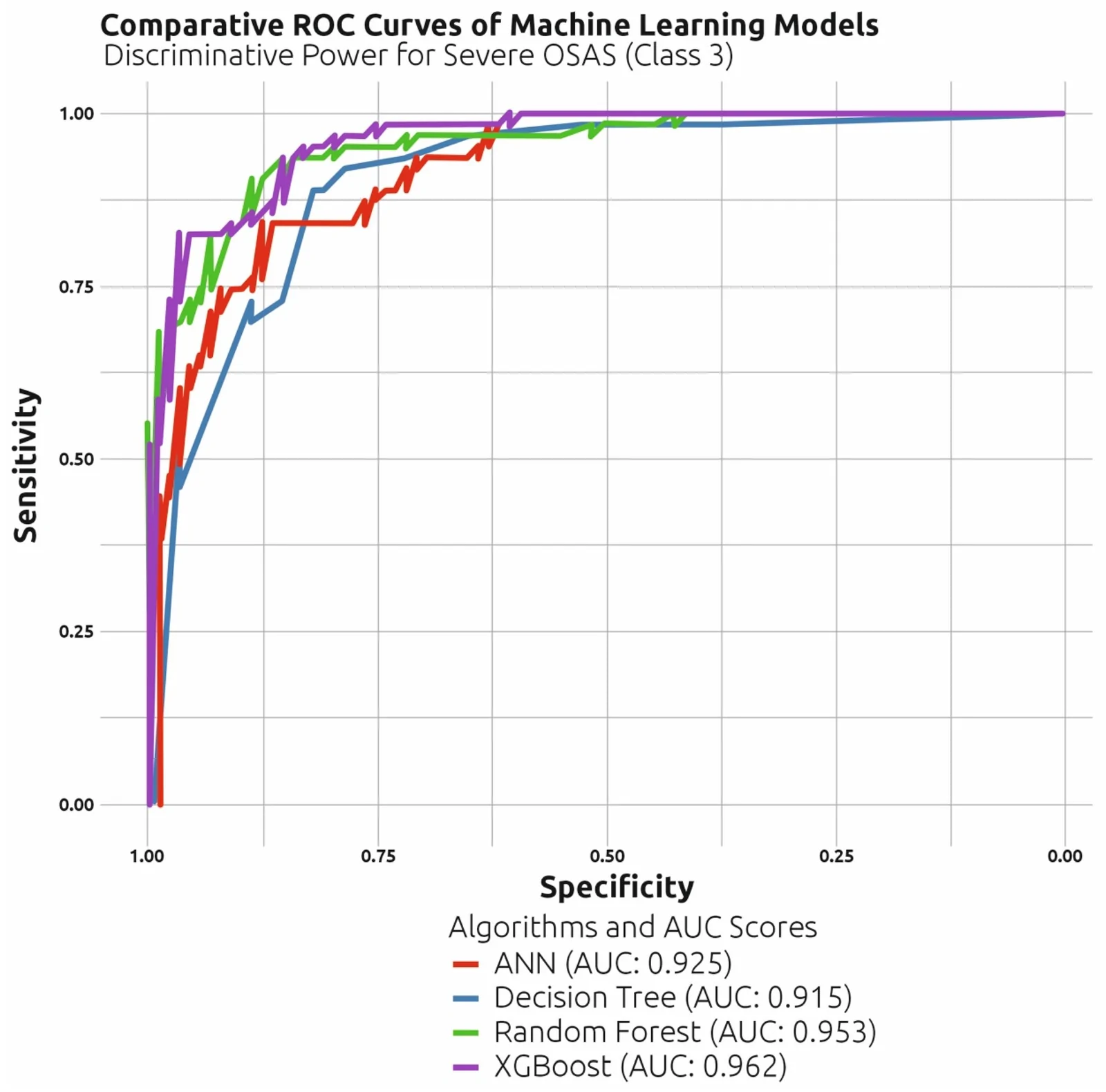
Comparison of ROC Curves for Machine Learning Models in the Diagnosis of Severe OSA (Class 3).

In Figure 5 and Figure 6, the discriminatory performance of the artificial neural network (ANN), decision tree, random forest and XGBoost models in the diagnosis of severe OSAS (Class 3) is compared using ROC curves. All models demonstrated a high level of classification success, with area under the curve (AUC) values ranging from 0.915 to 0.962.

The highest performance was achieved by the XGBoost model, with an AUC value calculated as 0.962. This result demonstrates that the XGBoost model possesses near-perfect discriminatory power in distinguishing patients with severe OSAS from other groups. The XGBoost model was followed by the Random Forest model (AUC = 0.953), which also exhibited a very high classification performance.

Although the ANN model demonstrated strong discriminatory performance with an AUC value of 0.925, it lagged behind tree-based ensemble learning methods. The lowest performance was observed in the Decision Tree model (AUC = 0.915). However, this value is still well above acceptable thresholds, indicating that the model is successful in classifying severe OSAS.

Upon examining the overall appearance of the ROC curves, it can be seen that the curves for the XGBoost and Random Forest models, in particular, lie closer to the top left corner of the graph. This indicates that these models are capable of achieving both high sensitivity and high specificity at different threshold values. Our findings suggest that machine learning-based algorithms may demonstrate superior performance compared to single decision tree and artificial neural network models in the prediction of severe OSAS.

## 4. Discussion

In this study, the role of the Sleep Disturbance Ratio (SDR) a new biomarker derived from polysomnography data in the severity classification of OSAS was evaluated, and the performance of various machine learning algorithms was compared. The main findings of the study can be summarised under four main headings. Firstly, it was found that ensemble-based algorithms, particularly the Random Forest and XGBoost models, demonstrated higher performance in OSAS severity classification. Secondly, the Epworth Sleepiness Scale (ESS) emerged as the most dominant variable across all models. Thirdly, although SDR did not show significant differences between OSAS severity classes in univariate analysis, it emerged as a complementary biomarker associated with the likelihood of severe OSAS in model-based explainable AI analyses. Finally, the increased likelihood of severe OSAS in areas where low mean oxygen saturation co-occurs with high SDR values indicates that OSAS should be assessed not only in terms of respiratory event frequency but also in conjunction with hypoxic load and disrupted sleep architecture.

Although the AHI has been used as the primary measure in the diagnosis and severity classification of OSAS for many years, the current literature emphasises that the AHI does not fully reflect the clinical heterogeneity of the condition [4,14]. Significant differences may be observed in terms of symptom burden, cardiovascular risk, neurocognitive impairment and quality of life among patients with similar AHI values. Consequently, the assessment of non-AHI parameters such as hypoxic burden, desaturation patterns, arousal index and sleep architecture is becoming increasingly important [15,16,17]. Recent evidence suggests that AHI alone does not adequately reflect the heterogeneous physiological burden of OSA, and complementary metrics have been increasingly advocated to improve disease characterization [18,19]. The development of the SDR in our study is consistent with this ‘beyond AHI’ approach. The SDR has been designed as a new composite parameter capable of reflecting the neurophysiological burden of OSAS by quantitatively expressing the imbalance between non-restorative and restorative sleep stages.

In our study, whilst the Random Forest model achieved the highest accuracy rate, the XGBoost model demonstrated the highest AUC value for multi-class classification. Furthermore, in the ROC analysis for the diagnosis of severe OSAS, the fact that XGBoost’s AUC value was 0.962 and that of the Random Forest model was 0.953 indicates that ensemble-based algorithms are highly effective in distinguishing severe OSAS from other classes. This finding is consistent with recent studies reporting that tree-based methods such as XGBoost, LightGBM, Random Forest and similar approaches demonstrate high performance in OSAS classification [20,21,22,23]. Thanks to their ability to model the complex, non-linear relationships between clinical, anthropometric and polysomnographic variables, ensemble methods can produce more stable results compared to individual decision trees or classical artificial neural network models.

It is noteworthy that the artificial neural network model demonstrated lower accuracy and F1-score in our study compared to other models. This may be related to factors such as the size of the dataset, class distribution, hyperparameter optimisation and the number of variables. Whilst deep learning models generally demonstrate superior performance on large-scale datasets, ensemble models such as Random Forest and XGBoost often provide more balanced and interpretable results on medium-sized tabular clinical datasets [20,21]. Therefore, the superiority of tree-based models in our study can be regarded as a methodologically expected finding. Overall, our findings are in agreement with recent state-of-the-art machine learning studies demonstrating that tree-based ensemble algorithms provide robust performance for OSA severity prediction. However, unlike previous studies that primarily relied on conventional polysomnographic or respiratory parameters, our study introduces SDR as an interpretable sleep architecture-derived biomarker and evaluates its contribution using explainable artificial intelligence techniques. This complementary approach extends current machine learning frameworks by incorporating quantitative information on sleep architecture rather than relying solely on respiratory event frequency.

One of the key findings of our study is that the ESS score had the highest variable importance value across all models. The strong correlation between the ESS and AHI supports the view that subjective daytime sleepiness is closely related to the severity of OSAS. Although the ESS is a practical, low-cost and feasible assessment tool in clinical practice, its use as a diagnostic test on its own is insufficient. However, the fact that it achieves a high importance score in machine learning models suggests that it may make a meaningful contribution to algorithmic classification when symptom burden is assessed alongside PSG parameters. This finding supports the view that OSAS is not solely characterised by objective respiratory events, but that clinical symptoms also constitute a significant part of the disease burden [24,25]. Given the dominant influence of ESS, we performed an additional sensitivity analysis to evaluate the independent contribution of the remaining variables. After excluding ESS from the feature set, oxygenation-related variables remained the strongest predictors across all machine learning models, whereas the Sleep Disturbance Ratio (SDR) consistently retained moderate-to-high feature importance. These findings suggest that the contribution of SDR is not solely driven by the inclusion of ESS but rather reflects complementary objective physiological information related to sleep architecture. This analysis further supports the potential value of SDR as an adjunctive biomarker for OSA severity assessment.

The correlation pattern of the SDR parameter is physiologically meaningful. The fact that SDR shows a strong positive correlation with N2 sleep and a strong negative correlation with N3 sleep suggests that this parameter reflects an increase in light sleep and a loss of deep sleep. In OSAS, recurrent respiratory events, micro-awakenings and oxygen desaturations can disrupt sleep continuity, leading to a reduction in N3 and REM sleep and an increase in superficial sleep stages such as N1/N2 [26,27]. Therefore, these findings support the physiological rationale underlying SDR in the context of OSA. Although SDR was not found to be statistically significant in univariate analyses, it demonstrates that, when evaluating new biomarkers, machine learning approaches—which can reveal complex interactions between variables should be considered alongside traditional statistical methods. At this point, explainable AI analyses reinforce the original contribution of our study. Partial dependency plots have shown a slight but consistent upward trend in the likelihood of severe OSAS with an increase in the SDR value. Similarly, in the risk map illustrating the interaction between SDR and mean oxygen saturation, it was observed that the combination of low saturation and high SDR increases the likelihood of severe OSAS. This finding suggests that SDR, when evaluated alongside hypoxic load parameters, may better reflect the multidimensional nature of OSAS. The literature has also reported that hypoxic load may better reflect cardiovascular outcomes and disease burden than AHI [15,16,17]. Our study extends this approach with a new biomarker based on sleep architecture. While previous studies have investigated individual sleep-stage characteristics, sleep fragmentation, hypoxic burden, and arousal-related parameters as complementary dimensions of OSA severity, these variables are generally evaluated separately. To the best of our knowledge, no previous study has proposed a single polysomnography-derived biomarker that quantitatively integrates light sleep (N1 + N2) and restorative sleep (N3 + REM) into a unified measure for OSA assessment. SDR was developed to address this gap by providing a simple, interpretable, and physiologically meaningful index of sleep architecture disruption that can be readily incorporated into explainable machine learning models and future multidimensional phenotyping strategies [4,5,23]. Our findings are also supported by recent studies demonstrating that OSA is characterized not only by recurrent respiratory events but also by profound alterations in sleep architecture, including reduced slow-wave sleep, disrupted REM sleep, and increased sleep fragmentation. These changes have been associated with impaired cognitive function, excessive daytime sleepiness, and adverse cardiometabolic outcomes, emphasizing the importance of evaluating sleep architecture alongside conventional respiratory indices [7,25].

The clinical effects of OSAS are not limited solely to the number of apnoeas and hypopnoeas. The depth of hypoxia, duration of desaturation, sleep fragmentation and loss of restorative sleep may play a significant role in the cardiometabolic and neurocognitive outcomes of the condition [25,26]. Previous experimental studies have demonstrated that sleep fragmentation itself can produce excessive daytime sleepiness and neurobehavioral impairment even in the absence of substantial sleep deprivation, highlighting its independent physiological importance [28]. Therefore, the development of parameters that summarise sleep architecture, such as the SDR, may contribute to a more comprehensive assessment of OSAS severity. In particular, the increased likelihood of severe OSAS associated with a combination of a high SDR and low oxygen saturation highlights the need for a combined assessment of respiratory and neurophysiological impairment.

The explainable artificial intelligence approach used in our study is also clinically significant. One of the fundamental issues with the use of machine learning models in healthcare is that, due to the ‘black box’ nature of the models, decision-making processes are not sufficiently understood by clinicians [29,30]. Partial dependence analysis demonstrated a non-linear relationship between SDR and the predicted probabilities of OSA severity classes. Increasing SDR was generally associated with a higher predicted probability of severe OSA across the principal range of observed SDR values, although this relationship was not monotonic. In our study, the dominant effect of ESS, the contribution of oxygenation parameters and the complementary role of SDR have been demonstrated using interpretable methods. In this respect, the study not only presents a high-performance classification model but also renders the model’s behaviour physiologically interpretable.

However, caution should be exercised when interpreting the SDR. The fact that it does not show a significant difference in univariate analysis indicates that the SDR cannot directly distinguish between classes using a classical statistical approach. However, as machine learning models take into account interactions between variables and non-linear relationships, it is evident that the SDR can make a limited but meaningful contribution when used in conjunction with other parameters. This demonstrates that traditional statistical analyses and machine learning approaches are not alternatives to one another, but rather complementary.

Although SDR was developed and evaluated in patients with OSA, we do not propose it as an OSA-specific diagnostic index or as a replacement for the Apnoea–Hypopnoea Index (AHI). Similar alterations in sleep architecture may occur in several other sleep disorders and clinical conditions. Rather, SDR should be regarded as a quantitative marker of sleep fragmentation that complements conventional respiratory indices. In OSA, recurrent respiratory events and arousals disrupt normal sleep architecture by increasing light sleep and reducing restorative sleep. Accordingly, SDR captures an important physiological consequence of OSA rather than the obstructive respiratory events themselves. Its clinical value is likely to be greatest when interpreted alongside established respiratory indices, such as AHI and oxygenation parameters, rather than as a standalone measure. Future studies comparing patients with OSA and other sleep disorders associated with sleep fragmentation, such as insomnia, periodic limb movement disorder, and central sleep apnea, are warranted to determine the disease specificity and broader clinical applicability of SDR. Importantly, the value of SDR lies not in replacing established respiratory metrics, but in complementing them by capturing sleep architecture disruption in a single quantitative parameter that is readily interpretable in both conventional statistical analyses and explainable artificial intelligence frameworks.

This study has certain limitations. Firstly, the study has a retrospective, single-centre design. Consequently, multicentre and prospective validation studies are required to assess the generalisability of the results to different populations. Secondly, the models have not been tested in an external validation cohort. In addition, no suitable publicly available polysomnography dataset containing all variables required for SDR calculation and model development was available for external validation. Future studies should evaluate the proposed model using independent multicenter and publicly available datasets to confirm its reproducibility and generalizability. Thirdly, the SDR was calculated solely on the basis of sleep stage percentages derived from PSG, and its association with long-term clinical outcomes such as cardiovascular events, neurocognitive outcomes or treatment response has not been assessed. Fourthly, the dominant influence of the ESS on the model’s decision-making process may have reduced the relative contribution of objective polysomnographic variables, including SDR. Although a sensitivity analysis excluding ESS demonstrated that SDR retained meaningful feature importance, further validation in models based exclusively on objective physiological parameters is warranted to better define its independent clinical value. In future studies, it would be beneficial to evaluate SDR in conjunction with hypoxic load, the arousal index, total sleep time, sleep efficiency and post-treatment changes.

## 5. Conclusions

In conclusion, this study has demonstrated that ensemble-based machine learning algorithms, such as XGBoost and Random Forest, perform well in the severity classification of OSAS. Whilst ESS emerged as the strongest predictive variable, the newly developed SDR parameter, although not a significant classifier on its own, has attracted attention as a complementary biomarker associated with a high probability of severe OSAS in explainable AI analyses. Future multicentre and prospective validation studies are required to establish the value of SDR as a biomarker suitable for clinical use and to assess its contribution to a more accurate and personalised phenotyping of OSAS, going beyond the traditional AHI-based classification.

## Figures and Tables

**Table 1:** Performance Comparison of Machine Learning Models for OSA Severity Classification.

Model	Accuracy	Cohen’s Kappa	F1-Score	Multiclass AUC
Decision Tree (DT)	0.645	0.507	0.635	0.831
Random Forest (RF)	0.750	0.645	0.712	0.882
XGBoost	0.730	0.621	0.714	0.895
Artificial Neural Network (ANN)	0.625	0.480	0.597	0.849

**Table 2:** (A) Normalized Feature Importance Rankings Across Machine Learning Algorithms. (B) Sensitivity Analysis: Normalized Feature Importance Rankings (Excluding ESS).

Feature	Decision Tree	Random Forest	**XGBoost**
**(A)**			
**Epworth Sleepiness Scale (ESS)**	**100.00**	**100.00**	**100.00**
Percentage of Sleep Time with SpO_2_ 80–90%	61.07	43.07	13.78
Minimum Oxygen Saturation	67.52	38.24	7.21
Mean Oxygen Saturation	42.41	25.64	7.81
**Sleep Disturbance Ratio (SDR**)	6.50	23.61	10.60
Waist Circumference	26.03	25.09	7.12
Age	16.58	24.53	8.63
Body Mass Index (BMI)	2.18	23.42	7.78
**(B)**			
Percentage of Sleep Time with SpO_2_ 80–90%	**100.00**	**100.00**	**100.00**
Minimum Oxygen Saturation	86.82	89.74	80.64
Waist Circumference	82.53	59.57	37.30
**Sleep Disturbance Ratio (SDR)**	40.37	54.99	38.15
Age	20.91	57.05	40.62
Body Mass Index (BMI)	35.88	56.22	28.08
Mean Oxygen Saturation	55.66	50.99	25.11

## Data Availability

The raw data supporting the conclusions of this article will be made available by the authors upon reasonable request, subject to privacy and ethical restrictions related to patient data.
